# Supplementary material for: Molecular Epidemiologic and Geo-Spatial Characterization of Staphylococcus aureus Cultured from Skin and Soft Tissue Infections from United States-Born and Immigrant Patients Living in New York City
Source: Antibiotics (Basel). 2023 Oct 14;12(10):1541. doi: 10.3390/antibiotics12101541 (PMC10604313; doi:10.3390/antibiotics12101541)
Supplement: Supplementary file 1 [file antibiotics-12-01541-s001.zip › antibiotics-2590841-supplementary.pdf]

Table S1. Population Characteristics Variable Descriptions

**Gender**	Female, male
**Race**	Black, white, other ()
**Ethnicity**	Hispanic, Non-Hispanic
**Age**	< 19, 19-45, 45-65, and > 65 years old
**Marital Status**	Married, single
**Education**	Highest degree obtained<br>High school diploma or below, some college, Bachelor's or above
**Healthcare Insurance**	Public, private, none, other ()
**Self-Rated Health**	Self-reported overall health<br>Poor, fair, good (includes very good and excellent)
**Income**	Total combined family income for the past 12 months<br>≥ $40,000, < $40,000
**First Time Infection**	Is this a first-time infection?<br>Yes, No
**Crowding Life Environment**	Do they live, work, or spend time in a crowded environment, such as a bedroom with at least one other person, dormitory, prison, daycare center, nursing home, contact sports, or international travel in the past month?<br>Yes, No
**Healthcare Exposure**	Have they worked or spent time in a healthcare facility or correctional facility in the past month?<br>Yes, No
**Contact Animal**	Have they worked or spent time with animals (including pets) in the past month?<br>Yes, No
**Had Wounds**	Have they had surgery or a wound in the past month?<br>Yes, No
**Social Network**	Does anyone in their household have a risk factor for *S. aureus* infection, such as recent surgery, work in the healthcare field, or recent SSTI in the past month?<br>Yes, No
**Household Crowding**	Number of people (excluding newborns) that live in a household<br><2 people, ≥2 People
**Personal Hygiene**	Do they share personal hygiene products (towels, unwashed clothes, razors) with another person or re-wear clothing?<br>Yes, No
**Washing Hands**	How many times a day do they wash their hands?<br>< 10 times/day, > 10 times/day

Table S2. MRSA v MSSA in all study population.

	MRSA (N=67)	MSSA (N=60)	Total (N=127)	P-value
**Gender, n (%)**				0.10[1]
Female	32 (47.8%)	20 (33.3%)	52 (40.9%)	
Male	35 (52.2%)	40 (66.7%)	75 (59.1%)	
**Race, n (%)**				0.98[1]
Black	15 (36.6%)	15 (37.5%)	30 (37.0%)	
White	12 (29.3%)	11 (27.5%)	23 (28.4%)	
Other Race	14 (34.1%)	14 (35.0%)	28 (34.6%)	
Missing	26	20	46	
**Ethnicity, n (%)**				0.78[1]
Hispanic	43 (67.2%)	33 (64.7%)	76 (66.1%)	
Non-Hispanic	21 (32.8%)	18 (35.3%)	39 (33.9%)	
Missing	3	9	12	
**Age (Years), n (%)**				0.37[1]
< 19	6 (9.0%)	4 (6.7%)	10 (7.9%)	
19-45	43 (64.2%)	31 (51.7%)	74 (58.3%)	
45-65	16 (23.9%)	22 (36.7%)	38 (29.9%)	
> 65	2 (3.0%)	3 (5.0%)	5 (3.9%)	
**Birthplace, n (%)**				0.11[1]
US Born	47 (70.1%)	34 (56.7%)	81 (63.8%)	
Non-US Born	20 (29.9%)	26 (43.3%)	46 (36.2%)	
**Marital Status, n (%)**				0.77[1]
Couple	24 (36.4%)	20 (33.9%)	44 (35.2%)	
Single	42 (63.6%)	39 (66.1%)	81 (64.8%)	
Missing	1	1	2	
**Education, n (%)**				0.43[1]
High School or lower	41 (62.1%)	43 (72.9%)	84 (67.2%)	
College	15 (22.7%)	9 (15.3%)	24 (19.2%)	
Bachelor or higher	10 (15.2%)	7 (11.9%)	17 (13.6%)	
Missing	1	1	2	
**Health Insurance, n (%)**				0.68[1]
Private or Other	14 (21.2%)	10 (16.9%)	24 (19.2%)	
Public (Medicare or Medicaid)	39 (59.1%)	34 (57.6%)	73 (58.4%)	
None	13 (19.7%)	15 (25.4%)	28 (22.4%)	
Missing	1	1	2	
**Health Quality, n (%)**				0.79[1]
Good	44 (69.8%)	38 (64.4%)	82 (67.2%)	
Fair	16 (25.4%)	17 (28.8%)	33 (27.0%)	
Poor	3 (4.8%)	4 (6.8%)	7 (5.7%)	
Missing	4	1	5	

**Income, n (%)**				0.58[1]
≥$40,000	5 (10.0%)	6 (13.6%)	11 (11.7%)	
< $40,000	45 (90.0%)	38 (86.4%)	83 (88.3%)	
Missing	17	16	33	
**First Time Infection, n (%)**				0.58[1]
No	18 (28.6%)	14 (24.1%)	32 (26.4%)	
Yes	45 (71.4%)	44 (75.9%)	89 (73.6%)	
Missing	4	2	6	
**Crowding Life Environment, n (%)**				0.03[1]
Yes	46 (76.7%)	34 (57.6%)	80 (67.2%)	
No	14 (23.3%)	25 (42.4%)	39 (32.8%)	
Missing	7	1	8	
**Healthcare Exposure, n (%)**				0.80[1]
Yes	21 (34.4%)	22 (36.7%)	43 (35.5%)	
No	40 (65.6%)	38 (63.3%)	78 (64.5%)	
Missing	6	0	6	
**Animal Contact, n (%)**				0.08[1]
Yes	26 (41.9%)	44 (73.3%)	80 (65.6%)	
No	36 (58.1%)	16 (26.7%)	42 (34.4%)	
Missing	5	0	5	
**Had Wounds, n (%)**				0.08[1]
Yes	16 (25.4%)	24 (40.0%)	40 (32.5%)	
No	47 (74.6%)	36 (60.0%)	83 (67.5%)	
Missing	4	0	4	
**Social Network, n (%)**				0.40[1]
Yes	15 (24.6%)	11 (18.3%)	26 (21.5%)	
No	46 (75.4%)	49 (81.7%)	95 (78.5%)	
Missing	6	0	6	
**Household Crowding, n (%)**				0.94[1]
<2 People	17 (30.9%)	16 (30.2%)	33 (30.6%)	
>2 People	38 (69.1%)	37 (69.8%)	75 (69.4%)	
Missing	12	7	19	
**Personal Hygiene, n (%)**				0.06[1]
Not Sharing	29 (52.7%)	18 (34.6%)	47 (43.9%)	
Sharing	26 (47.3%)	34 (65.4%)	60 (56.1%)	
Missing	12	8	20	
**Hand Washing, n (%)**				0.11[1]
<10 Times/Day	40 (63.5%)	46 (76.7%)	86 (69.9%)	
>10 Times/Day	23 (36.5%)	14 (23.3%)	37 (30.1%)	
Missing	4	0	4	
***S. aureus* Genotype, n (%)**				0.0001[1]
USA300	39 (58.2%)	12 (20.0%)	51 (40.2%)	

Non-USA300	28 (41.8%)	48 (80.0%)	76 (59.8%)	
**mecA Gene from *S. aureus* Wound, n (%)**				<.0001[1]
Positive	60 (90.9%)	0 (0.0%)	60 (48.8%)	
Negative	6 (9.1%)	53 (93.0%)	59 (48.0%)	
Not Determined	0 (0.0%)	4 (7.0%)	4 (3.3%)	
Missing	1	3	4	
**ACME Gene from *S. aureus* Wound, n (%)**				<.0001[1]
Negative	27 (40.9%)	51 (89.5%)	78 (63.4%)	
Type I	38 (57.6%)	2 (3.5%)	40 (32.5%)	
Type III	1 (1.5%)	0 (0.0%)	1 (0.8%)	
Not Determined	0 (0.0%)	4 (7.0%)	4 (3.3%)	
Missing	1	3	4	
**SCCmec Gene from *S. aureus* Wound, n (%)**				<.0001[1]
Negative	1 (1.5%)	1 (1.8%)	2 (1.6%)	
IVa	52 (78.8%)	0 (0.0%)	52 (42.3%)	
IVb	1 (1.5%)	0 (0.0%)	1 (0.8%)	
IVc	3 (4.5%)	0 (0.0%)	3 (2.4%)	
IVg	1 (1.5%)	0 (0.0%)	1 (0.8%)	
IVh	1 (1.5%)	0 (0.0%)	1 (0.8%)	
Not Determined	5 (7.6%)	56 (98.2%)	61 (49.6%)	
Novel Type	2 (3.0%)	0 (0.0%)	2 (1.6%)	
Missing	1	3	4	
**PVL Gene from *S. aureus* Wound, n (%)**				0.0001[1]
Positive	50 (75.8%)	20 (35.1%)	70 (56.9%)	
Negative	16 (24.2%)	33 (57.9%)	49 (39.8%)	
Not Determined	0 (0.0%)	4 (7.0%)	4 (3.3%)	
Missing	1	3	4	

[1]Chi-Square p-value;

Table S3. USA300 v non-USA300 in all study population.

	USA300 (n=51)	Non-USA300 (n=76)	Total (n=127)	P-value
**Gender, n (%)**				0.68[1]
Female	22 (43.1%)	30 (39.5%)	52 (40.9%)	
Male	29 (56.9%)	46 (60.5%)	75 (59.1%)	
**Race, n (%)**				0.84[1]
Black	13 (40.6%)	17 (34.7%)	30 (37.0%)	
White	9 (28.1%)	14 (28.6%)	23 (28.4%)	
Other Race	10 (31.3%)	18 (36.7%)	28 (34.6%)	
Missing	19	27	46	
**Ethnicity, n (%)**				0.88[1]
Hispanic	32 (65.3%)	44 (66.7%)	76 (66.1%)	
Non-Hispanic	17 (34.7%)	22 (33.3%)	39 (33.9%)	
Missing	2	10	12	
**Age (Years), n (%)**				0.91[1]
< 19	3 (5.9%)	7 (9.2%)	10 (7.9%)	
19-45	31 (60.8%)	43 (56.6%)	74 (58.3%)	
45-65	15 (29.4%)	23 (30.3%)	38 (29.9%)	
> 65	2 (3.9%)	3 (3.9%)	5 (3.9%)	
**Birthplace, n (%)**				0.04[1]
US Born	38 (74.5%)	43 (56.6%)	46 (35.2%)	
Non-US Born	13 (25.5%)	33 (43.4%)	81 (63.8%)	
**Marital Status, n (%)**				0.54[1]
Couple	16 (32.0%)	28 (37.3%)	44 (35.2%)	
Single	34 (68.0%)	47 (62.7%)	81 (64.8%)	
Missing	1	1	2	
**Education, n (%)**				0.40[1]
High School or lower	33 (66.0%)	51 (68.0%)	84 (67.2%)	
College	12 (24.0%)	12 (16.0%)	24 (19.2%)	
Bachelor or higher	5 (10.0%)	12 (16.0%)	17 (13.6%)	
Missing	1	1	2	
**Health Insurance, n (%)**				0.52[1]
Private or Other	12 (24.0%)	12 (16.0%)	24 (19.2%)	
Public (Medicare or Medicaid)	28 (56.0%)	45 (60.0%)	73 (58.4%)	
None	10 (20.0%)	18 (24.0%)	28 (22.4%)	
Missing	1	1	2	
**Health Quality, n (%)**				0.58[1]
Good	31 (63.3%)	51 (69.9%)	82 (67.2%)	
Fair	14 (28.6%)	19 (26.0%)	33 (27.0%)	
Poor	4 (8.2%)	3 (4.1%)	7 (5.7%)	

Missing	2	3	5	
**Income, n (%)**				0.66[1]
≥$40,000	4 (10.0%)	7 (13.0%)	11 (11.7%)	
< $40,000	36 (90.0%)	47 (87.0%)	83 (88.3%)	
Missing	11	22	33	
**First Time Infection, n (%)**				0.66[1]
No	14 (28.6%)	18 (25.0%)	32 (26.4%)	
Yes	35 (71.4%)	54 (75.0%)	89 (73.6%)	
Missing	2	4	6	
**Crowding Life Environment, n (%)**				0.10[1]
Yes	35 (76.1%)	45 (61.6%)	80 (67.2%)	
No	11 (23.9%)	28 (38.4%)	39 (32.8%)	
Missing	5	3	8	
**Healthcare Exposure, n (%)**				0.51[1]
Yes	15 (31.9%)	28 (37.8%)	43 (35.5%)	
No	32 (68.1%)	46 (62.2%)	78 (64.5%)	
Missing	4	2	6	
**Animal Contact, n (%)**				0.03[1]
Yes	22 (45.8%)	20 (27.0%)	42 (34.4%)	
No	26 (54.2%)	54 (73.0%)	80 (65.6%)	
Missing	3	2	5	
**Had Wounds, n (%)**				0.25[1]
Yes	13 (26.5%)	27 (36.5%)	40 (32.5%)	
No	36 (73.5%)	47 (63.5%)	83 (67.5%)	
Missing	2	2	4	
**Social Network, n (%)**				0.30[1]
Yes	8 (16.7%)	18 (24.7%)	26 (21.5%)	
No	40 (83.3%)	55 (75.3%)	95 (78.5%)	
Missing	3	3	6	
**Household Crowding, n (%)**				0.18[1]
<2 People	10 (23.3%)	23 (35.4%)	33 (30.6%)	
>2 People	33 (76.7%)	42 (64.6%)	75 (69.4%)	
Missing	8	11	19	
**Personal Hygiene, n (%)**				0.10[1]
Not Sharing	23 (53.5%)	24 (37.5%)	47 (43.9%)	
Sharing	20 (46.5%)	40 (62.5%)	60 (56.1%)	
Missing	8	12	20	
**Hand Washing, n (%)**				0.92[1]
<10 Times/Day	34 (69.4%)	52 (70.3%)	86 (69.9%)	
>10 Times/Day	15 (30.6%)	22 (29.7%)	37 (30.1%)	
Missing	2	2	4	
**mecA Gene from *S. aureus* Wound, n (%)**				<0.0001[1]

Positive	39 (78.0%)	21 (28.8%)	60 (48.8%)	
Negative	11 (22.0%)	48 (65.8%)	59 (48.0%)	
Not Determined	0 (0.0%)	4 (5.5%)	4 (3.3%)	
Missing	1	3	4	
**ACME Gene from *S. aureus* Wound, n (%)**				<0.0001[1]
Negative	14 (28.0%)	64 (87.7%)	78 (63.4%)	
Type I	35 (70.0%)	5 (6.8%)	40 (32.5%)	
Type III	1 (2.0%)	0 (0.0%)	1 (0.8%)	
Not Determined	0 (0.0%)	4 (5.5%)	4 (3.3%)	
Missing	1	3	4	
**SCCmec Gene from *S. aureus* Wound, n (%)**				<0.0001[1]
Negative	1 (2.0%)	1 (1.4%)	2 (1.6%)	
IVa	38 (76.0%)	14 (19.2%)	52 (42.3%)	
IVb	0 (0.0%)	1 (1.4%)	1 (0.8%)	
IVc	1 (2.0%)	2 (2.7%)	3 (2.4%)	
IVg	0 (0.0%)	1 (1.4%)	1 (0.8%)	
IVh	0 (0.0%)	1 (1.4%)	1 (0.8%)	
Not Determined	10 (20.0%)	51 (69.9%)	61 (49.6%)	
Novel Type	0 (0.0%)	2 (2.7%)	2 (1.6%)	
Missing	1	3	4	
**PVL Gene from *S. aureus* Wound, n (%)**				<0.0001[1]
Positive	41 (82.0%)	29 (39.7%)	70 (56.9%)	
Negative	9 (18.0%)	40 (54.8%)	49 (39.8%)	
Not Determined	0 (0.0%)	4 (5.5%)	4 (3.3%)	
Missing	1	3	4	

[1]Chi-Square p-value

Table S4. Population Characteristics of patients with *S. aureus isolated* from wound cultures.

	MRSA				MSSA			
	**US Born (N=47)**	**Non-US Born (N=20)**	**Total (N=67)**	**P-value**	**US Born (N=34)**	**Non-US Born (N=26)**	**Total (N=61)**	**P-value**
**Gender, n (%)**				0.77[1]				0.85[1]
Female	23 (48.9%)	9 (45.0%)	32 (47.8%)		11 (32.4%)	9 (34.6%)	20 (33.3%)	
Male	24 (51.1%)	11 (55.0%)	35 (52.2%)		23 (67.6%)	17 (65.4%)	40 (66.7%)	
**Race, n (%)**				0.009[2]				0.24[1]
Black	15 (45.5%)	0 (0.0%)	15 (36.6%)		12 (46.2%)	3 (21.4%)	15 (37.5%)	
White	10 (30.3%)	2 (25.0%)	12 (29.3%)		7 (26.9%)	4 (28.6%)	11 (27.5%)	
Other	8 (24.2%)	6 (75.0%)	14 (34.1%)		7 (26.9%)	7 (50.0%)	14 (35.0%)	
Missing	14	12	26		8	12	20	
**Ethnicity, n (%)**				0.06[1]				0.04[1]
Hispanic	27 (60.0%)	16 (84.2%)	43 (67.2%)		16 (53.3%)	17 (81.0%)	33 (64.7%)	
Non-Hispanic	18 (40.0%)	3 (15.8%)	21 (32.8%)		14 (46.7%)	4 (19.0%)	18 (35.3%)	
Missing	2	1	3		4	5	9	
**Age, n (%)**				0.35[2]				0.11[2]
<19 Years	6 (12.8%)	0 (0.0%)	6 (9.0%)		4 (11.8%)	0 (0.0%)	4 (6.7%)	
19-45 Years	28 (59.6%)	15 (75.0%)	43 (64.2%)		15 (44.1%)	16 (61.5%)	31 (51.7%)	
45-65 Years	11 (23.4%)	5 (25.0%)	16 (23.9%)		12 (35.3%)	10 (38.5%)	22 (36.7%)	
> 65 Years	2 (4.3%)	0 (0.0%)	2 (3.0%)		3 (8.8%)	0 (0.0%)	3 (5.0%)	
**Marital Status, n (%)**				0.0006[1]				0.0003[1]
Couple	11 (23.4%)	13 (68.4%)	24 (36.4%)		5 (14.7%)	15 (60.0%)	20 (33.9%)	
Single	36 (76.6%)	6 (31.6%)	42 (63.6%)		29 (85.3%)	10 (40.0%)	39 (66.1%)	
Missing	0	1	1		0	1	1	
**Education, n (%)**				0.003[2]				0.14[2]
High School or Below	30 (63.8%)	11 (57.9%)	41 (62.1%)		24 (72.7%)	19 (73.1%)	43 (72.9%)	
College	14 (29.8%)	1 (5.3%)	15 (22.7%)		7 (21.2%)	2 (7.7%)	9 (15.3%)	

Bachelor or Above	3 (6.4%)	7 (36.8%)	10 (15.2%)		2 (6.1%)	5 (19.2%)	7 (11.9%)	
Missing	0	1	1		1	0	1	
**Health Insurance, n (%)**				0.001[1]				0.002[1]
Private or Other	10 (21.3%)	4 (21.1%)	14 (21.2%)		4 (11.8%)	6 (24.0%)	10 (16.9%)	
Public	33 (70.2%)	6 (31.6%)	39 (59.1%)		26 (76.5%)	8 (32.0%)	34 (57.6%)	
None	4 (8.5%)	9 (47.4%)	13 (19.7%)		4 (11.8%)	11 (44.0%)	15 (25.4%)	
Missing	0	1	1		0	1	1	
**Health Quality, n (%)**				0.25[2]				0.92[2]
Good	28 (63.6%)	16 (84.2%)	44 (69.8%)		22 (66.7%)	16 (61.5%)	38 (64.4%)	
Fair	13 (29.5%)	3 (15.8%)	16 (25.4%)		9 (27.3%)	8 (30.8%)	17 (28.8%)	
Poor	3 (6.8%)	0 (0.0%)	3 (4.8%)		2 (6.1%)	2 (7.7%)	4 (6.8%)	
Missing	3	1	4		1	0	1	
**Income, n (%)**				0.38[1]				0.81[1]
≥$40,000	3 (7.9%)	2 (16.7%)	5 (10.0%)		3 (12.5%)	3 (15.0%)	6 (13.6%)	
< $40,000	35 (92.1%)	10 (83.3%)	45 (90.0%)		21 (87.5%)	17 (85.0%)	38 (86.4%)	
Missing	9	8	17		10	6	16	
**First Time Infection, n (%)**				0.79[1]				0.16[1]
No	13 (29.5%)	5 (26.3%)	18 (28.6%)		10 (31.3%)	4 (15.4%)	14 (24.1%)	
Yes	31 (70.5%)	14 (73.7%)	45 (71.4%)		22 (68.8%)	22 (84.6%)	44 (75.9%)	
Missing	3	1	4		2	0	2	
**Crowding Life Environment, n (%)**				0.78[1]				0.11[1]
Yes	31 (75.6%)	15 (78.9%)	46 (76.7%)		16 (48.5%)	18 (69.2%)	34 (57.6%)	
no	10 (24.4%)	4 (21.1%)	14 (23.3%)		17 (51.5%)	8 (30.8%)	25 (42.4%)	
Missing	6	1	7		1	0	1	
**Healthcare Exposure, n (%)**				0.37[1]				0.41[1]
No	26 (61.9%)	14 (73.7%)	40 (65.6%)		20 (58.8%)	18 (69.2%)	38 (63.3%)	

Yes	16 (38.1%)	5 (26.3%)	21 (34.4%)		14 (41.2%)	8 (30.8%)	22 (36.7%)	
Missing	5	1	6		0	0	0	
**Contact Animal, n (%)**				0.27[1]				0.58[1]
No	23 (53.5%)	13 (68.4%)	36 (58.1%)		24 (70.6%)	20 (76.9%)	44 (73.3%)	
Yes	20 (46.5%)	6 (31.6%)	26 (41.9%)		10 (29.4%)	6 (23.1%)	16 (26.7%)	
	4	1	5		0	0	0	
**Had Wounds, n (%)**				0.25[1]				0.20[1]
No	31 (70.5%)	16 (84.2%)	47 (74.6%)		18 (52.9%)	18 (69.2%)	36 (60.0%)	
Yes	13 (29.5%)	3 (15.8%)	16 (25.4%)		16 (47.1%)	8 (30.8%)	24 (40.0%)	
Missing	3	1	4					
**Social Network, n (%)**				0.28[1]				0.41[1]
No	30 (71.4%)	16 (84.2%)	46 (75.4%)		29 (85.3%)	20 (76.9%)	49 (81.7%)	
Yes	12 (28.6%)	3 (15.8%)	15 (24.6%)		5 (14.7%)	6 (23.1%)	11 (18.3%)	
Missing	5	1	6		0	0	0	
**Household Crowding, n (%)**				0.08[1]				0.57[1]
< 2 People	15 (37.5%)	2 (13.3%)	17 (30.9%)		10 (33.3%)	6 (26.1%)	16 (30.2%)	
>2 People	25 (62.5%)	13 (86.7%)	38 (69.1%)		20 (66.7%)	17 (73.9%)	37 (69.8%)	
Missing	7	5	12		4	3	7	
**Personal Hygiene, n (%)**				0.06[1]				0.98[1]
Not Sharing	18 (45.0%)	11 (73.3%)	29 (52.7%)		10 (34.5%)	8 (34.8%)	18 (34.6%)	
Sharing	22 (55.0%)	4 (26.7%)	26 (47.3%)		19 (65.5%)	15 (65.2%)	34 (65.4%)	
Missing	7	5	12		5	3	8	
**Hand Washing, n (%)**				0.97[1]				0.51[1]
<10 times/day	28 (63.6%)	12 (63.2%)	40 (63.5%)		25 (73.5%)	21 (80.8%)	46 (76.7%)	
>10 times/day	16 (36.4%)	7 (36.8%)	23 (36.5%)		9 (26.5%)	5 (19.2%)	14 (23.3%)	
Missing	3	1	4		0	0	0	

[1]Chi-Square p-value; [2]Fisher Exact p-value

Table S5. Population Characteristics based on birthplace and USA300 Genotype.

	USA300				Non-USA300			
	US Born (N=38)	Non-US Born (N=13)	Total (N=51)	P-value	US Born (N=43)	Non-US Born (N=33)	Total (N=76)	P-value
**Gender, n (%)**				0.69[1]				0.99[1]
Female	17 (44.7%)	5 (38.5%)	22 (43.1%)		17 (39.5%)	13 (39.4%)	30 (39.5%)	
Male	21 (55.3%)	8 (61.5%)	29 (56.9%)		26 (60.5%)	20 (60.6%)	46 (60.5%)	
**Race, n (%)**				0.03[2]				0.12[2]
Black	13 (50.0%)	0 (0.0%)	13 (40.6%)		14 (42.4%)	3 (18.8%)	17 (34.7%)	
White	7 (26.9%)	2 (33.3%)	9 (28.1%)		10 (30.3%)	4 (25.0%)	14 (28.6%)	
Other	6 (23.1%)	4 (66.7%)	10 (31.3%)		9 (27.3%)	9 (56.3%)	18 (36.7%)	
Missing	12	7	19		10	17	27	
**Ethnicity, n (%)**				0.50[2]				0.01[2]
Hispanic	22 (61.1%)	10 (76.9%)	32 (65.3%)		21 (53.8%)	23 (85.2%)	44 (66.7%)	
Non-Hispanic	14 (38.9%)	3 (23.1%)	17 (34.7%)		18 (46.2%)	4 (14.8%)	22 (33.3%)	
Missing	2	0	2		4	6	10	
**Age, n (%)**				0.93[2]				0.02[2]
<19 Years	3 (7.9%)	0 (0.0%)	3 (5.9%)		7 (16.3%)	0 (0.0%)	7 (9.2%)	
19-45 Years	22 (57.9%)	9 (69.2%)	31 (60.8%)		21 (48.8%)	22 (66.7%)	43 (56.6%)	
45-65 Years	11 (28.9%)	4 (30.8%)	15 (29.4%)		12 (27.9%)	11 (33.3%)	23 (30.3%)	
> 65 Years	2 (5.3%)	0 (0.0%)	2 (3.9%)		3 (7.0%)	0 (0.0%)	3 (3.9%)	
**Marital Status, n (%)**				0.01[2]				0.0001[1]

Couple	8 (21.1%)	8 (66.7%)	16 (32.0%)		8 (18.6%)	20 (62.5%)	28 (37.3%)	
Single	30 (78.9%)	4 (33.3%)	34 (68.0%)		35 (81.4%)	12 (37.5%)	47 (62.7%)	
Missing	0	1	1		0	1	1	
**Education, n (%)**				0.03[2]				0.03[2]
High School or Below	24 (63.2%)	9 (75.0%)	33 (66.0%)		30 (71.4%)	21 (63.6%)	51 (68.0%)	
College	12 (31.6%)	0 (0.0%)	12 (24.0%)		9 (21.4%)	3 (9.1%)	12 (16.0%)	
Bachelor or Above	2 (5.3%)	3 (25.0%)	5 (10.0%)		3 (7.1%)	9 (27.3%)	12 (16.0%)	
Missing	0	1	1		1	0	1	
**Healthcare Insurance, n (%)**				0.0004[2]				0.002[1]
Private or other	9 (23.7%)	3 (25.0%)	12 (24.0%)		5 (11.6%)	7 (21.9%)	12 (16.0%)	
Public	26 (68.4%)	2 (16.7%)	28 (56.0%)		33 (76.7%)	12 (37.5%)	45 (60.0%)	
none	3 (7.9%)	7 (58.3%)	10 (20.0%)		5 (11.6%)	13 (40.6%)	18 (24.0%)	
Missing	0	1	1		0	1	1	
**Self-Rated Health, n (%)**				0.68[2]				0.83[2]
Fair	11 (29.7%)	3 (25.0%)	14 (28.6%)		11 (27.5%)	8 (24.2%)	19 (26.0%)	
Good	22 (59.5%)	9 (75.0%)	31 (63.3%)		28 (70.0%)	23 (69.7%)	51 (69.9%)	
Poor	4 (10.8%)	0 (0.0%)	4 (8.2%)		1 (2.5%)	2 (6.1%)	3 (4.1%)	
Missing	1	1	2		3	0	3	
**Income**, n (%)				0.21[2]				1.00[2]
=>$40,000	2 (6.5%)	2 (22.2%)	4 (10.0%)		4 (12.9%)	3 (13.0%)	7 (13.0%)	
< $40,000	29 (93.5%)	7 (77.8%)	36 (90.0%)		27 (87.1%)	20 (87.0%)	47 (87.0%)	

Missing	7	4	11		12	10	22	
**First Time Infection, n (%)**				0.47[2]				0.49[1]
No	12 (32.4%)	2 (16.7%)	14 (28.6%)		11 (28.2%)	7 (21.2%)	18 (25.0%)	
Yes	25 (67.6%)	10 (83.3%)	35 (71.4%)		28 (71.8%)	26 (78.8%)	54 (75.0%)	
Missing	1	1	2		4	0	4	
**Crowding Life Environment, n (%)**				0.70[2]				0.20[1]
Yes	25 (73.5%)	10 (83.3%)	35 (76.1%)		22 (55.0%)	23 (69.7%)	45 (61.6%)	
No	9 (26.5%)	2 (16.7%)	11 (23.9%)		18 (45.0%)	10 (30.3%)	28 (38.4%)	
Missing	4	1	5		3	0	3	
**Healthcare Exposure, N (%)**				0.73[2]				0.23[1]
Yes	12 (34.3%)	3 (25.0%)	15 (31.9%)		18 (43.9%)	10 (30.3%)	28 (37.8%)	
No	23 (65.7%)	9 (75.0%)	32 (68.1%)		23 (56.1%)	23 (69.7%)	46 (62.2%)	
Missing	3	1	4		2	0	2	
**Contact Animal, n (%)**				0.74[1]				0.12[1]
Yes	16 (44.4%)	6 (50.0%)	22 (45.8%)		14 (34.1%)	6 (18.2%)	20 (27.0%)	
No	20 (55.6%)	6 (50.0%)	26 (54.2%)		27 (65.9%)	27 (81.8%)	54 (73.0%)	
Missing	2	1	3		2	0	2	
**Had Wounds**, n (%)				0.47[2]				0.14[1]
Yes	11 (29.7%)	2 (16.7%)	13 (26.5%)		18 (43.9%)	9 (27.3%)	27 (36.5%)	
No	26 (70.3%)	10 (83.3%)	36 (73.5%)		23 (56.1%)	24 (72.7%)	47 (63.5%)	

Missing	1	1	2		2	0	2	
**Social Network**, n (%)				1.00[2]				0.54[1]
Yes	6 (16.7%)	2 (16.7%)	8 (16.7%)		11 (27.5%)	7 (21.2%)	18 (24.7%)	
No	30 (83.3%)	10 (83.3%)	40 (83.3%)		29 (72.5%)	26 (78.8%)	55 (75.3%)	
Missing	2	1	3		3	0	3	
**Household Crowding**, n (%)				0.41[2]				0.13[1]
<2 People	9 (27.3%)	1 (10.0%)	10 (23.3%)		16 (43.2%)	7 (25.0%)	23 (35.4%)	
>2 People	24 (72.7%)	9 (90.0%)	33 (76.7%)		21 (56.8%)	21 (75.0%)	42 (64.6%)	
Missing	5	3	8		6	5	11	
**Personal Hygiene, n (%)**				0.29[2]				0.44[1]
Not Sharing	16 (48.5%)	7 (70.0%)	23 (53.5%)		12 (33.3%)	12 (42.9%)	24 (37.5%)	
Sharing	17 (51.5%)	3 (30.0%)	20 (46.5%)		24 (66.7%)	16 (57.1%)	40 (62.5%)	
Missing	5	3	8		7	5	12	
**Washing Hands, n (%)**				0.30[2]				0.92[1]
<10 times/day	24 (64.9%)	10 (83.3%)	34 (69.4%)		29 (70.7%)	23 (69.7%)	52 (70.3%)	
>10 times/day	13 (35.1%)	2 (16.7%)	15 (30.6%)		12 (29.3%)	10 (30.3%)	22 (29.7%)	
Missing	1	1	2		2	0	2	

[1]Chi-Square p-value; [2]Fisher Exact p-value

Table S6. Molecular characteristics of wound identified as *Staphylococcus aureus* by birthplace and type.

	MRSA				MSSA			
	US born (N=47)	Non-US born (N=20)	Total (N=67)	P-value[1]	US born (N=34)	Non-US born (N=26)	Total (N=60)	P-value[1]
**mecA Gene from *S. aureus* Wound, n (%)**				1.00				0.62
Positive	42 (91.3%)	18 (90.0%)	60 (90.9%)		0 (0.0%)	0 (0.0%)	0 (0.0%)	
Negative	4 (8.7%)	2 (10.0%)	6 (9.1%)		27 (87.1%)	25 (92.6%)	52 (89.7%)	
Not Determined	0 (0.0%)	0 (0.0%)	0 (0.0%)		3 (9.7%)	1 (3.8%)	4 (7.0%)	
Missing	1	0	1		3	0	3	
**ACME Gene from *S. aureus* Wound, n (%)**				0.25				0.44
Negative	16 (34.8%)	11 (55.0%)	27 (40.9%)		26 (83.9%)	25 (96.2%)	51 (89.5%)	
Type I	29 (63.0%)	9 (45.0%)	38 (57.6%)		2 (6.5%)	0 (0.0%)	2 (3.5%)	
Type III	1 (2.2%)	0 (0.0%)	1 (1.5%)		0 (0.0%)	0 (0.0%)	0 (0.0%)	
Not Determined					3 (9.7%)	1 (3.8%)	4 (7.0%)	
Missing	1	0	1		3	0	3	
**SCCmec Gene from *S. aureus* Wound, n (%)**				0.90				1.00
Negative	0 (0.0%)	1 (5.0%)	1 (1.5%)		1 (3.2%)	0 (0.0%)	1 (1.8%)	
Iva	35 (76.1%)	17 (85.0%)	52 (78.8%)		0 (0.0%)	0 (0.0%)	0 (0.0%)	
IVb	1 (2.2%)	0 (0.0%)	1 (1.5%)		0 (0.0%)	0 (0.0%)	0 (0.0%)	
IVc	2 (4.3%)	1 (5.0%)	3 (4.5%)		0 (0.0%)	0 (0.0%)	0 (0.0%)	
IVg	1 (2.2%)	0 (0.0%)	1 (1.5%)		0 (0.0%)	0 (0.0%)	0 (0.0%)	
IVh	1 (2.2%)	0 (0.0%)	1 (1.5%)		0 (0.0%)	0 (0.0%)	0 (0.0%)	
Not Determined	4 (8.7%)	1 (5.0%)	5 (7.6%)		30 (96.8%)	26 (100.0%)	56 (98.2%)	
Novel Type	2 (4.3%)	0 (0.0%)	2 (3.0%)		0 (0.0%)	0 (0.0%)	0 (0.0%)	
Missing	1	0	1		3	0	3	
**PVL Gene from *S. aureus* Wound, n (%)**				0.54				0.77
Positive	36 (78.3%)	14 (70.0%)	50 (75.8%)		11 (35.5%)	9 (34.6%)	20 (35.1%)	
Negative	10 (21.7%)	6 (30.0%)	16 (24.2%)		17 (54.8%)	16 (61.5%)	33 (57.9%)	
Not Determined	0 (0.0%)	0 (0.0%)	0 (0.0%)		3 (9.7%)	1 (3.8%)	4 (7.0%)	
Missing	1	0	1		3	0	3	

[1]Fisher Exact p-value

Table S7. Molecular characteristics for genotype by birthplace and *Staphylococcus aureus*.

	USA300				Non-USA300			
	**US Born (N=38)**	**Non-US Born (N=13)**	**Total (N=51)**	**P-value**	**US Born (N=43)**	**Non-US Born (N=33)**	**Total (N=76)**	**P-value**
**mecA Gene from *S. aureus* Wound, n (%)**				0.44[2]				0.73[2]
**Positive**	30 (81.1%)	9 (69.2%)	39 (78.0%)		12 (30.0%)	9 (27.3%)	21 (28.8%)	
**Negative**	7 (18.9%)	4 (30.8%)	11 (22.0%)		25 (62.5%)	23 (69.7%)	48 (65.8%)	
**Not Determined**	0 (0.0%)	0 (0.0%)	0 (0.0%)		3 (7.5%)	1 (3.0%)	4 (5.5%)	
**Missing**	1	0	1		3	0	3	
**ACME Gene from *S. aureus* Wound , n (%)**				0.23[2]				0.76[2]
Negative	8 (21.6%)	6 (46.2%)	14 (28.0%)		34 (85.0%)	30 (90.9%)	64 (87.7%)	
Not Determined	0 (0.0%)	0 (0.0%)	0 (0.0%)		3 (7.5%)	1 (3.0%)	4 (5.5%)	
Type I	28 (75.7%)	7 (53.8%)	35 (70.0%)		3 (7.5%)	2 (6.1%)	5 (6.8%)	
Type III	1 (2.7%)	0 (0.0%)	1 (2.0%)		0 (0.0%)	0 (0.0%)	0 (0.0%)	
Missing	1	0	1		3	0	3	
**SCCmec Gene from *S. aureus* Wound, n (%)**				0.69[2]				0.70[2]
Negative	1 (2.7%)	0 (0.0%)	1 (2.0%)		0 (0.0%)	1 (3.0%)	1 (1.4%)	
Iva	29 (78.4%)	9 (69.2%)	38 (76.0%)		6 (15.0%)	8 (24.2%)	14 (19.2%)	
IVb	0 (0.0%)	0 (0.0%)	0 (0.0%)		1 (2.5%)	0 (0.0%)	1 (1.4%)	
IVc	1 (2.7%)	0 (0.0%)	1 (2.0%)		1 (2.5%)	1 (3.0%)	2 (2.7%)	
IVg	0 (0.0%)	0 (0.0%)	0 (0.0%)		1 (2.5%)	0 (0.0%)	1 (1.4%)	
IVh	0 (0.0%)	0 (0.0%)	0 (0.0%)		1 (2.5%)	0 (0.0%)	1 (1.4%)	
Not Determined	6 (16.2%)	4 (30.8%)	10 (20.0%)		28 (70.0%)	23 (69.7%)	51 (69.9%)	
Novel Type	0 (0.0%)	0 (0.0%)	0 (0.0%)		2 (5.0%)	0 (0.0%)	2 (2.7%)	
Missing	1	0	1		3	0	3	
**PVL Gene from *S. aureus* Wound, n (%)**				0.21[2]				0.74[2]
Positive	32 (86.5%)	9 (69.2%)	41 (82.0%)		15 (37.5%)	14 (42.4%)	29 (39.7%)	

Negative	5 (13.5%)	4 (30.8%)	9 (18.0%)		22 (55.0%)	18 (54.5%)	40 (54.8%)
Not Determined	0 (0.0%)	0 (0.0%)	0 (0.0%)		3 (7.5%)	1 (3.0%)	4 (5.5%)
Missing	1	0	1		3	0	3

[1]Chi-Square p-value; [2]Fisher Exact p-value

Table S8. Ethnic community neighborhoods by race and ethnicity stratified by US-born and non-US born

	US Born					Non-US Born				
	Patient Race and Ethnicity					Patient Race and Ethnicity				
**Community Neighborhood**	Black (N=27)	White (N=17)	Other (N=15)	Hispanic[1] (N=43)	Missing (N=22)	Black (N=3)	White (N=6)	Other (N=13)	Hispanic[1] (N=33)	Missing (N=24)
Americas	0 (0.0%)	0 (0.0%)	2 (15.4%)	2 (4.9%)	0	0 (0.0%)	0 (0.0%)	1 (8.3%)	3 (9.4%)	2
Asia	0 (0.0%)	1 (5.9%)	0 (0.0%)	0 (0.0%)	0	0 (0.0%)	1 (16.7%)	0 (0.0%)	2 (6.3%)	2
Black	15 (55.6%)	5 (29.4%)	5 (38.5%)	9 (22.0%)	4	2 (100.0%)	1 (16.7%)	1 (8.3%)	3 (9.4%)	3
Europe	0 (0.0%)	0 (0.0%)	0 (0.0%)	0 (0.0%)	1	0 (0.0%)	1 (16.7%)	0 (0.0%)	0 (0.0%)	0
Hispanic	9 (33.3%)	4 (23.5%)	6 (46.2%)	26 (63.4%)	15	0 (0.0%)	3 (50.0%)	8 (66.7%)	19 (59.4%)	12
White	3 (11.1%)	7 (41.2%)	0 (0.0%)	4 (9.8%)	2	0 (0.0%)	0 (0.0%)	2 (16.7%)	5 (15.6%)	5
Missing	0	0	2	2	0	1	0	1	1	0

[1]Patients who reported Hispanic ethnicity are not excluded in race counts

Table S9. Distribution of ESRI tapestry segment name by methicillin susceptibility stratified by US-born and non-US born.

			US Born			Non-US Born		
**Life Mode Group**	**Neighborhood Segment Name**	**Description**	**MRSA N (%)**	**MSSA N (%)**	**p-value**	**MRSA N (%)**	**MSSA N (%)**	**p-value**
Upscale Avenues	Enterprising Professionals	White, College Degree	1 (2.2%)	0 (0%)	0.32	0 (0%)	0 (0.0%)	0.29
	Pacific Heights	Asian/Pacific Islander, College Degree	0 (0%)	0 (0%)		1 (5.3%)	0 (0.0%)	
Uptown Individuals	Laptops and Lattes	White, College Degree	0 (0%)	0 (0%)		0 (0%)	1 (4.0%)	
	Trendsetters	White, College Degree	0 (0%)	0 (0%)		0 (0%)	1 (4.0%)	
Middle Ground	City Lights	White, College Degree	2 (4.4%)	0 (0%)		0 (0%)	0 (0.0%)	
	Downtown Melting Pot	White/Asian / Pacific Islander, College Degree	5 (10.9%)	5 (15.2%)		2 (10.5%)	7 (28.0%)	
Senior Styles	Golden Years	White, College Degree	0 (0%)	0 (0%)		1 (5.3%)	0 (0.0%)	
	Social Security Set	White/Black, HS Diploma Only	0 (0%)	1 (3.0%)		0 (0%)	0 (0.0%)	
Midtown Singles	City Strivers	Black, HS Diploma Only	4 (8.7%)	6 (18.2%)		2 (10.5%)	3 (12.0%)	
Hometown	Family Foundations	Black, HS Diploma Only	0 (0%)	0 (0%)		1 (5.3%)	0 (0.0%)	
Next Wave	High Rise Renters	Hispanic, No HS Diploma	31 (67.4%)	21 (63.6%)		9 (47.4%)	8 (32.0%)	
	International Marketplace	Hispanic, No HS Diploma	3 (6.5%)	0 (0%)		1 (5.3%)	5 (20.0%)	

	Las Casas	Hispanic, No HS Diploma	0 (0%)	0 (0%)	1 (5.3%)	0 (0.0%)
	NeWest Residents	Hispanic, No HS Diploma	0 (0%)	0 (0%)	1 (5.3%)	0 (0.0%)
Missing	Unclassified	Insufficient data for classification	0	0	1	1
